# Hydroxychloroquine and dexamethasone in COVID-19: who won and who lost?

**DOI:** 10.1186/s12948-020-00132-7

**Published:** 2020-09-09

**Authors:** Claudio Ortolani, Elide A. Pastorello

**Affiliations:** 1 Istituto Allergologico Lombardo. Casa Di Cura Ambrosiana, Cesano Boscone, Milano, Italy; 2 Unit of Allergy and Immunology, Università Degli Studi Di Milano, ASST Grande Ospedale Metropolitano Niguarda, Milano, Italy

**Keywords:** COVID-19, SARS-CoV-2, Cytokine storm syndrome, ARDS, Hydroxychloroquine, Remdesivir, Dexamethasone, Lopinavir—ritonavir, Evidence based medicine

## Abstract

**Background:**

On June 30, 2020, the WHO reported over 10 millions of COVID-19 cases worldwide with over half a million deaths. In severe cases the disease progresses into an Acute Respiratory Distress Syndrome (ARDS), which in turn depends on an overproduction of cytokines (IL-6, TNFα, IL-12, IL-8, CCL-2 and IL1) that causes alveolar and vascular lung damage. Clearly, it is essential to find an immunological treatment that controls the “cytokine storm”. In the meantime, however, it is essential to have effective antiviral and anti-inflammatory drugs available immediately.

**Pharmacologic therapy for COVID-19:**

Hydroxychloroquine or chloroquine have been widely adopted worldwide for the treatment of SARS-CoV-2 pneumonia. However, the choice of this treatment was based on low quality of evidence, i.e. retrospective, non-randomized controlled studies. Recently, four large Randomized Controlled Trials (RCTs) have been performed in record time delivering reliable data: (1) the National Institutes of Health (NIH) RCT included 60 hospitals participating all over the world and showed the efficacy of remdesivir in reducing the recovery time in hospitalized adults with COVID-19 pneumonia; (2) three large RCTs already completed, for hydroxychloroquine, dexamethasone and Lopinavir and Ritonavir respectively. These trials were done under the umbrella of the 'Recovery' project, headed by the University of Oxford. The project includes 176 participating hospitals in the UK and was set up to verify the efficacy of some of the treatments used for COVID-19. These three ‘Recovery’ RCTs concluded definitely: (a) that treatment with hydroxychloroquine provides no benefits in patients hospitalized with COVID-19; (b) that treatment with dexamethasone reduced deaths by one-third in COVID-19 patients that were mechanically ventilated, and by one-fifth in patients receiving oxygen only; (c) that the combination of Lopinavir and Ritonavir is not effective in reducing mortality in COVID-19 hospitalized patients.

**Conclusions:**

The results of these four large RCTs have provided sound indications to doctors for the treatment of patients with COVID-19 and prompted the correction of many institutional provisions and guidelines on COVID-19 treatments (i.e. FDA, NIH, UK Health Service, etc.). Even though a definitive treatment for COVID-19 has not yet been found, large RCTs stand as the Gold Standards for COVID-19 therapy and offer a solid scientific base on which to base treatment decisions.

## Background

In many countries, the COVID-19 pandemic flared up rapidly bringing excellent hospitals and efficient national health systems to their knees. The biggest challenges were: dealing with a previously unknown disease, without effective drugs, and a global mortality of 5%. Current data (June 30, 2020) from the World Health Organization (WHO) leave no doubt about the severity of this pandemic: the number of COVID-19 cases totaled over 10 millions, worldwide, with over half a million deaths [1]. In severe cases, the disease progresses into interstitial pneumonia with ARDS [2–4], which in turn is largely due to a cytokine (IL-6, TNFα, IL-12, IL-8, CCL-2 and IL-1) overproduction which causes alveolar and vascular lung damage [5–8]. Presently, we do not have any specific antiviral, chemotherapeutic or vaccine measures, nor do we have an anti-inflammatory drug capable of fighting the 'cytokine storm'. Oxygen supplementation and assisted mechanical ventilation are the only two stages of care for respiratory failure during ARDS; both help keep the patient alive, but neither promotes healing.

## Pharmacologic therapy

In 2019, at the beginning of the SARS-CoV-2 pandemic, at least 4 anti-inflammatory and antiviral drugs were available and in use, with possible efficacy for COVID-19: hydroxychloroquine, corticosteroids, remdesivir and Lopinavir / Ritonavir.

### Chloroquine/hydroxychloroquine

The rationale for the use of chloroquine as a drug for COVID-19 was based on the demonstration of its strong antiviral effect on SARS-CoV in primate cell cultures [9]. The antiviral effect in vitro was related, first, to the known increase in the pH of endosomes that the virus uses for cell entry and, second, to an impairment of terminal glycosilation of the cellular receptor of SARS-CoV-2, angiotensin converting enzyme 2, which is the binding site for the DRB (Determinant Receptor Binding) of the spike glycoprotein of coronaviruses. Furthermore, it has been recently confirmed that chloroquine and, even more markedly, hydroxychloroquine have anti-SARS-CoV-2 activity in vitro cell cultures [10].

### Corticosteroids

In severe cases, COVID-19 is complicated by pneumonia, anatomically characterized by inflammatory alveolar infiltrates and vascular microthrombi. An exaggerated host immune response seems to be an important factor leading to clinical aggravation. These patients have very high inflammatory markers, such as C reactive protein, ferritin, IL-1 and IL-6. Therefore, in these cases, it is rational to try the efficacy of corticosteroids. At the beginning of 2020, however, the use of corticosteroids in COVID-19 was a controversial topic. The situation is well represented on the one hand by the contrary opinion of Russel et al. (2020) expressed in their comment published on Lancet [11]. The authors stated that there is no clinical data that indicates a net benefit from corticosteroids in the treatment of respiratory infections due to RSV, influenza, SARS-CoV, or MERS-CoV. Conversely, they stated that the available observational data suggested an increased mortality and secondary infection rates in influenza, an impaired clearance of SARS-CoV and MERS-CoV, and complications of corticosteroid therapy in survivors.

On the other hand, in another comment published on Lancet, Shang et al. [12] expressed a completely opposite opinion. This was based both on the results of a retrospective study on patients with severe SARS pneumonia, which demonstrated a reduction in mortality and hospitalization after treatment with moderate doses of corticosteroids, and, on the results of a study of over 2000 patients with severe H1N1 influenza pneumonia, in which a reduction in mortality was observed in patients with an Oxygen Index lower than 300 mm Hg [12]. Given the importance of resolving this disparity of opinion, it was essential to carry out randomized and controlled studies on a large sample of COVID-19 patients evaluating the results in relation to the severity of the disease.

#### Remdesivir

Among the available antiviral drugs, remdesivir was the most promising to be effective against SARS-CoV-2. This is a small-molecule, monophosphoramidate prodrug of a nucleotide analogue, that is intracellularly metabolized to an analogue of adenosine triphosphate that inhibits viral RNA dependent RNA polymerases (RdRp), which had demonstrated in vivo antiviral efficacy against Ebola virus in non-human primates [13]. Because its mechanism of action on viral RdRp and previous observations of its activity against filoviruses (e.g. Ebola) and coronaviruses (e.g. SARS-CoV and MERS-CoV) both in vitro and in various models of animal infection [14, 15], it was justified to evaluate its efficacy in COVID-19 in a polycentric RCT with a large case series.

#### Lopinavir / Ritonavir

Lopinavir is a protease inhibitor used to treat HIV infections. It is commercially associated with a subtherapeutic dose of ritonavir, which is a pharmacokinetic enhancer and inhibitor of the cytochrome P450 isoenzyme 3A4 resulting in inhibition of the metabolism of lopinavir and an increase in its pharmacological exposure. Protease is a key enzyme in coronavirus polyprotein processing and lopinavir and/or ritonavir (LPV / r) showed an antiviral effect against SARS-CoV-2 in vitro [16]. Previous observational studies of the efficacy of LPV / r treatment in COVID-19 have obtained conflicting results (positive or uncertain or negative) [17]. The only RCT on 199 COVID-19 patients found no difference between usual treatments and that with the addition of LPV / r [18]. Therefore it was justified to verify the efficacy of this drug through an RCT on a large sample of COVID-19 patients.

#### Clinical trials

One of the first European studies on COVID-19 treatments was conducted in Marseille, France. Prof. Didier Raoult and his team adopted an early drug treatment with hydroxychloroquine and azithromycin in COVID-19 patients with confirmed pneumonia. The same team of researchers had previously shown that the combination of these two drugs was effective against the SARS-CoV-2 virus both in vitro [19] and in vivo [20]. More than 3,700 COVID-19 patients were treated in Marseille with a protocol that included: early diagnosis, early isolation and early treatment, with 200 mg of oral hydroxychloroquine, three times daily for ten days and 500 mg of oral azithromicin on day 1 followed by 250 mg daily for the next four days respectively, for at least three days.The results of this treatment were described in a final, overall retrospective study [21], and consisted in reduced risks of death, transfer to the ICU or hospitalization, and a shorter viral shedding period, against modest side effects.

Unfortunately, since patients underwent a complete protocol, which included first of all the promptness of each intervention, it is not possible to know if the positive results obtained in COVID-19 patients were attributable only to the drugs, or to the entire protocol used.

So far, very few Randomized and Controlled Trials (RCTs) have been planned and performed for COVID-19, despite the great availability of patients and the urgency to demonstrate the effectiveness of the medications that could be used. On the other hand, several open, nonrandomized, studies have been carried out on a limited number of patients, but they have been irrelevant for the purpose of deciding which therapy to use in COVID-19 patients.

Recently, however, four large RCTs, performed in record time and aimed at demonstrating the effectiveness of some drugs in COVID-19, have finally brought some reliable results. The NIH study on remdesivir is an excellent example of what can be done even in emergency conditions [22]. The study was completed in about a month, with the participation of 60 hospitals, including 45 in the USA, 8 in Denmark, 5 in Great Britain, 4 in Greece, 3 in Germany, 2 in Korea, 2 in Mexico and one each in Spain and Singapore. The study enrolled 1,063 COVID-19 pneumonia patients, 538 of whom were assigned to the treatment with remdesivir and 521 to placebo. The results of this study showed the effectiveness of remdesivir in treating COVID-19 patients: the drug was superior to placebo in reducing the recovery time in hospitalized adults with COVID-19 pneumonia (p < 0.001). Mortality was also reduced in patients treated with remdesivir however, this result did not differ significantly from the controls. Even though the effects of the drug on SARS-CoV-19 are modest, since remdesivir is non-specific for this virus, the study results are reliable and the use of remdesivir’s in COVID-19 patients is justifed. However, the most important consequence of the NIH RCT’s results is that it has shown that an analog antiviral inhibitor nucleotide, such as remdesivir, is effective against SARS-CoV-2. This opened the way for researching and testing other drugs in this category to find an effective cure for COVID-19.

In order to correctly establish the effectiveness of some treatments in COVID-19, the 'Recovery' project was created with the intent of performing a series of randomized studies  based on a very large sample size. The project involves 176 hospitals of the National Health Service of England, Scotland, Wales and Northern Ireland. The first study completed, on June 5, 2020, concerned the effectiveness of hydroxychloroquine in COVID-19 [23, 24]. During the course of the study, an independent data monitoring committee reviewed every two weeks the results achieved, so that the study could be stopped when the latter were statistically significant and no longer modifiable by a further increase in the number of cases. The committee discontinued the study when it was clear that there was no benefit from the hydroxychloroquine treatment in hospitalized COVID-19 patients. On June 5, 2020, Prof. Peter Horby and Prof. Martin Landray, chief investigators of the Recovery Trial, announced that the data was considered conclusive. The RCT included a total of 1,542 hospitalized patients who had been treated with hydroxychloroquine and 3,132 control patients who had received normal treatment (without hydroxychloroquine). Patients were eligible for the study if they had clinically suspected or laboratory confirmed SARS-CoV-2 infection, and no significant risk for participating in the hydroxychloroquine arm. Patients with known prolonged electrocardiograph QTc interval were ineligible for the trial. Patients allocated to hydroxychloroquine sulfate received a loading dose of 800 mg at zero and 6 h, followed by 400 mg starting at 12 h after the initial dose and then every 12 h for the next 9 days or until discharge. Participants and local study staff were not blinded to the allocated treatment. At randomization, 17% were receiving invasive mechanical ventilation or extracorporeal membrane oxygenation, 60% were receiving oxygen only (with or without non-invasive ventilation), and 24% were receiving neither. No significant difference was found between the two treatment arms in relation to the 28-day mortality rate (p = 0.10), and no beneficial effects on the duration of the hospital stay or other outcomes were also reported. Similar results were seen across all five pre-specified subgroups: i.e. 1- Age, 2- Sex, 3- Days since symptoms’ onset, 4- Respiratory support at randomization (e.g. no oxygen received, oxygen only and, invasive mechanical ventilation), 5- Baseline risks. Horby and Landray concluded that these data convincingly exclude any significant mortality advantage of hydroxychloroquine in hospitalized COVID-19 patients.

Eleven days after announcing the negative results of the RCT on the effectiveness of hydroxychloroquine in COVID-19, Landray and Horby, announced the positive results of the “RCT Recovery” on the effectiveness of dexamethasone in COVID-19 [25, 26]. The study protocol involved the enrollment of 11,500 patients from over 176 hospitals within the UK National Health Service. Patients were eligible for the trial if they had clinically suspected or laboratory confirmed SARS-CoV-2 infection and no medical history of being at substantial risk to participate in the trial. Pregnant or breast- feeding women were eligible. Of the 11,303 patients who underwent randomization from March 19 to June 8, 2020, 6425 underwent randomization to receive either oral or intravenous dexamethasone (2104 patients) -at a dose of 6 mg once daily for up to 10 days—or to receive usual care alone (4321 patients). The mean (± SD) age of the patients in this study was 66.1 (± 15.7) years, and 36% of the patients were female. At randomization, 16% were receiving invasive mechanical ventilation or extracorporeal membrane oxygenation, 60% were receiving oxygen only (with or without non-invasive ventilation), and 24% were receiving neither. In the dexamethasone group, 95% of the patients received at least one dose of the drug and the median duration of treatment was 7 days.

The primary outcome of the study was to evaluate the difference in mortality rate, calculated at 28 days from the start of treatment, between subjects treated with dexamethasone + usual therapy and those treated with usual therapy alone. Dexamethasone treatment reduced deaths by one-third in mechanically ventilated patients (p = 0.0003) and by one-fifth in patients receiving oxygen only (p = 0.0021). However, in patients who did not need any breathing support, there was no difference in mortality in the subjects treated with dexamethasone compared to controls (p = 0.14).

Finally, on June 29, 2020, Landray and Horby announced the completion of a third Recovery trial on the effectiveness of the Lopinavir-Ritonavir combination in Covid-19 patients. This study foresaw the enrollment of 11,800 patients in 176 NHS hospitals in the UK but, as in the RCTs “Recovery”, patient enrollment was terminated when the results reached statistical significance [27]. In this study, 1,596 patients were randomized to Lopinar-Ritonivar and 3,376 to usual care alone. No significant differences were found in the primary endpoint of 28-day mortality (p = 0.58), and there was also no evidence of any beneficial effects on reduction of the risk of progression to mechanical ventilation or length of hospital stay. Among these patients, 70% required oxygen alone, and 26% did not require any respiratory intervention, while only 4% of enrolled patients required mechanical ventilation. This is due to the difficulty of administering a drug by mouth in patients under mechanical ventilation. Therefore, in the most severe patients the results still require confirmation.

## Change of provisions

The results of the two “Recovery” RCTs on hydroxychloroquine and dexamethasone have rapidly changed the panorama of COVID-19 treatments, as well as previous therapeutic provisions. In fact, on June 15, 2020, the *Food & Drugs Administration (FDA)* revoked the authorization to use hydroxychloroquine and chloroquine, as an emergency treatment for COVID-19 [28], while the United Kingdom National Health Service has already announced that the standard of care for COVID-19 patients will now include dexamethasone [29].

After six months of a generalized and devastating pandemic, a correct scientific experimentation has provided us with two important results on the anti-inflammatory treatment of COVID-19: 1) a lack of evidence of the efficacy of hydroxychloroquine, and, 2) evidence that dexamethasone reduces mortality by one third in the most serious form of the disease, i.e. ARDS. These results are good for improving the perspectives of the treatment for the SARS-CoV-2 viral inflammation, but they are more than excellent for providing solid references in order to amend previous errors. They reaffirm the irreplaceable value of Evidence-Based Medicine (EBM), which—as we know—is the only pathway to rely on in diagnostics and therapy, and which was neglected during the panic-stricken emergency. This non-compliance has been maintained by many clinical doctors and officials from public health agencies, who authorized the use of a drug for COVID-19, which was then administered in almost all patients, despite the absence of any sound scientific evidence, and who, at the same time, recommended not to use corticosteroids in COVID-19 pneumonia. The Guidelines, published by WHO and the U.S. National Institutes of Health, also recommended not to use corticosteroids in COVID-19, because of a generally accepted concern that, by reducing the immune response, their use could facilitate the worsening of the SARS-CoV-2 infection or the occurrence of secondary infections.

It may be useful, for the purpose of a broader understanding, to analyze the chapter on corticosteroids in the WHO document of 13 March 2020 *“Clinical management of severe acute respiratory infection (SARI) when COVID-19 disease is suspected. Interim guidance. World Health Organization”* [30]. The statement: *“Do not routinely administer systemic corticosteroids for the treatment of viral pneumonia outside of clinical trials*” was supported by some remarks. Remark 1 cited a number of systematic reviews, which however had selected only observational clinical studies that addressed the efficacy and side effects of the corticosteroid treatment of viral pneumonia from SARS, H1N1 influenza virus and MERS virus, but not from SARS-CoV-2 virus [31–34]. Remark 2 made a conditional recommendation for corticosteroids for all patients with sepsis (including septic shock) [35].

In the revised WHO document of 27 May 2020 [36], in addition to the aforementioned citations, in Remark 1 a systematic review was added on the effectiveness of corticosteroids in viral pneumonia [37]. In this review, 10 observational studies were selected (5 for SARS, 4 for COVID-19 and one for MERS) and one RCT (for SARS [38]). There was no evidence of efficacy of the corticosteroid treatment in these cases of viral pneumonia, but an increase in side effects was reported. The authors of the systematic review, however, concluded that “*because of a preponderance of observational studies in the dataset and selection and publication biases our conclusions, especially regarding SARS-CoV-2, need confirmation in a randomized clinical trial*”. Moreover, the latest WHO document also cites a recent study by Villar et al. [39]: “*In addition, a recent trial reported that corticosteroids may reduce mortality in moderate-severe ARDS*”. Unfortunately, it was not stressed that, at that time, the study by Villar et al. was the only RTC available on the efficacy of a corticosteroid which demonstrated the efficacy of dexamethasone in ARDS (not COVID-19) with high quality evidence. Perhaps the results of this RCT could have casted some doubts to the authors of the WHO guidelines about the negative conclusions on the effectiveness of corticosteroids in ARDS from viral infections reported in previous observational studies. Villar et al.'s multicenter, randomized, controlled trial was conducted in 17 Intensive Care Units in Spanish Hospitals from 2013 to 2018, and enrolled 277 patients with moderate to severe confirmed ARDS, all receiving mechanical ventilation. The study concluded that the early administration of dexamethasone reduced the duration of mechanical ventilation and overall mortality in patients with moderate / severe ARDS (p < 0.0001), while the percentage of adverse events did not significantly differ between the dexamethasone group and the control arms.

On June 25, 2020, the NIH updated the Pharmacologic Intervention section of their COVID-19 Treatment Guidelines by including panel recommendations to use remdesivir in patients who are on mechanical ventilation or extracorporeal membrane oxygenation, and to use dexamethasone in patients who are mechanically ventilated and in those who require supplemental oxygen but are not mechanically ventilated [40].

## Conclusions

After these four large RCTs, it is legitimate to ask ourselves: “Who won and who lost?”.

EBM -which in turn must be based essentially on RCT clinical trials- won. All recommendations not based on EBM lost. Because they are not exempt from personal opinions, even if expressed in “bona fides” given the situation. Regarding the efficacy of glucocorticoids in COVID-19, an EBM evaluation might have simply stated that there is no evidence for or against treatment with these drugs in general, and that, since february 2020, there is a high quality evidence that dexamethasone reduces the number of days of mechanical ventilation and the mortality in ARDS, i.e. by Villar et al. [39].

Those who made exceptional efforts to organize in a very short time the execution of controlled and randomized trials, with a high participation of patients, aimed at verifying the efficiency of the treatments currently available for COVID-19—namely NIH and “Recovery”—won. All those who described clinical observations on small numbers of patients without control cases, not only lost, but also confused everybody. In particular, all those who too quickly accepted the widespread opinion that hydroxychloroquine was effective in COVID-19, without first thoroughly researching if any sound scientific evidence supported this claim, lost. Also, many researchers who, having planned excellent RCTs in one location only or in few centers, were forced to terminate them early due to the lack of patients, with the consequence that the data obtained were insufficient, unfortunately lost.

A new phase of clinical trials to evaluate the efficacy of drugs for COVID-19 is currently opening up. Indeed, a wide variety of drug screening assays, to explore the potential effectiveness on SARS-CoV-2 of old and new molecules, are currently underway. For example, in non-human primates and in cell cultures in vitro, there are at least 21 drugs, selected from a chemical library that contains nearly 12,000 drugs, that have a good chance of being effective against SARS-CoV-2 [41]. Our experience with the recent pandemic taught us that the most efficient and fastest way to verify the clinical efficacy of new antiviral drugs is: first, the realization of RCTs with wide participation of patients hospitalized for COVID-19, and second (and above all), the mandatory involvement of both international and national governmental health agencies, to organize, manage and control these necessary large trials. These Institutions in turn will have to make extensive use of the structural and organizational resources of the National Health Services present in their countries, as the RECOVERY Collaborative Group in the UK taught us.

Although many mistakes were made during choices of treatments for Covid-19 patients during this pandemic, the lesson that derives provides us with a reassuring message for the future: rigorous scientific research will find anti-SARS-CoV-2 treatments and EBM will be able to confirm which of these treatments is effective and safe.

## Data Availability

Not applicable.

## Abbreviations

ARDS: Acute Respiratory Distress Syndrome
CCL-2: C–C Motif ChemoKine Ligand 2
COVID-19: Coronavirus Disease-2019
DRB: Determinant Receptor Binding
EBM: Evidence Based Medicine
WHO: World Health Organization
FDA: Food and Drug Administration of USA Government
H1N1: A influence virus, subtype H1N1
ICU: Intensive Care Unit
IL-1: Interleukin 1
IL-6: Interleukin 6
IL-8: Interleukin 8
IL-12: Interleukin 12
LPV/r: Lopinavir/Ritonavir
MERS: Middle East Respiratory Syndrome
NHS: National Health Service of United Kingdoms
NIH: National Institutes of Health of USA Government
RCT: Randomized Controlled Trial
RdRp: RNA dependent RNA polymerase
SARI: Severe Acute Respiratory Infection
SARS: Severe Acute Respiratory Syndrome
SARS-CoV-2: Severe Acute Respiratory Syndrome Coronavirus 2
TNFα: Tumor Necrosis Factor alfa
